# Influence of short video usage on adolescent’s learning strategies: investigation on teenagers in HeBei North China

**DOI:** 10.1038/s41598-025-30713-y

**Published:** 2025-12-01

**Authors:** Liu Ziye, Zhao Yuying, Mao Ziyuan, Han Zhimin, Li Xinhua

**Affiliations:** 1 Medical Technology College, HeBei Medical University, No.316 Zhong’shan E Rd, Shijiazhuang, 050017 HeBei China; 2 Department of Cardiology, No.980 Hospital of PLA Joint Logistics Support Force, No.398 Zhong’shan W Rd, Shijiazhuang, 050082 HeBei China; 3 Academic Affairs Office, The Second Hospital of HeBei Medical University, No.215 Heping W Rd, Shijiazhuang, 050000 HeBei China

**Keywords:** Short-video usage, Digital screen time, Learning strategies, Adolescent, Education, Health care, Psychology, Psychology

## Abstract

The proliferation of digital devices has led to increased screen time among adolescents, raising concerns about its impact on health, development, and academic performance. This study aimed to investigate the current state of digital screen time associated with short-videos among adolescents, analyze learning strategies across different demographic groups, and evaluate the influence of short-videos on adolescents’ daily lives and learning strategies. A quantitative validation study was conducted with 4515 participants from 18 schools in HeBei province. Data were collected through a questionnaire that included demographic information, reasons for watching short-videos, and Zhang Yeheng’s Learning Strategy Scale. Statistical analysis was performed using SPSSAU software, with non-parametric tests to compare groups. After removing invalid questionnaires, 3028 valid responses were analyzed. Middle school students scored higher in learning strategies than high school students. Participants who owned mobile phones and used these devices primarily on weekends had higher learning strategy scores. Conversely, increased daily screen time was associated with lower learning strategy scores. Short-video usage has a complex impact on adolescent learning strategies. While providing informational resources, excessive use may distract and impair learning outcomes. Collaborative efforts from families, schools, and society are needed to guide healthy short-video usage for adolescents’ learning development.

## Introduction

The pervasive incorporation of digital media into the daily lives of adolescents has precipitated substantial changes in learning behaviors, especially in light of the swift proliferation of short-form video platforms^1,2^. These short-videos, generally characterized as video content with a duration of up to 5 min—though definitions vary, ranging from a few seconds to 20 min—are distributed via computers or mobile devices. The rise of short-videos has brought about new opportunities and challenges for students. On one hand, short-video content can be a source of learning, with many educational short-videos available that can supplement classroom learning. On the other hand, the highly engaging and often entertainment- oriented nature of short-videos may also distract students from their studies and disrupt their established learning strategies. For instance, students might be tempted to spend excessive time “scrolling” through short-video feeds, which could lead to less time dedicated to traditional learning activities such as reading textbooks or doing homework.​.

The attributes of being concise, entertaining, and personalized align with the public’s demand for fragmented information in the Internet age, resulting in rapid proliferation of short-videos. Although these platforms provide access to educational content, there is evidence indicating that excessive short video may hinder cognitive self-regulation and academic performance^3^. Excessive screen time is linked to poor academic engagement, with studies showing that many children and adolescents spend too much time on screens, negatively affecting their physical, psychological, and cognitive development^4^. This screen time may reduce participation in activities that enhance learning based on the theory of Resource Model of Self-Control. Additionally, frequent media switching can overload working memory, which is vital for cognitive function, leading to decreased efficiency and undermining metacognitive strategies essential for problem-solving and learning^5^. Adolescents, having weaker inhibitory control than adults, are particularly vulnerable to digital distractions. In contrast to traditional long-form videos, which typically necessitate extended viewing times and are predominantly produced by professional entities (such as feature-length films, television series, and documentaries that generally exceed 30 min in duration), short-videos are predominantly user-generated and more readily accessible. These short-videos can be swiftly created and disseminated, aligning with the fast-paced lifestyles of contemporary individuals, particularly the youth. Platforms such as TikTok and Douyin facilitate the effortless production, editing, and uploading of short-videos. Moreover, the sophisticated algorithm-based recommendation systems employed by these platforms enable the rapid dissemination of popular short-videos to a broad audience.

Despite the growing popularity of short-videos among students, there is a lack of in-depth research on how this usage pattern intersects with their learning strategies. This research attempts to fill this gap by providing a comprehensive analysis of students’ short-video usage and its implications for their learning strategies.

### Theoretical framework

The Resource Model of Self-Control, proposed by Baumeister et al. in 1998, is a key model in social psychology. It suggests that self-control relies on finite resources that are depleted with use. Initially, it was believed that only high-demand tasks, like resisting bad habits, led to significant depletion. However, recent studies indicate that any effortful task can deplete these self-control resources^6^. Algorithmic content delivery reinforces continuous scrolling, fragmenting sustained focus. The design of short-video platforms and the nature of the content they deliver can significantly impact users’ ability to maintain sustained attention. Drawing on this theory, we posit that allocating finite cognitive resources to short-video consumption depletes the capacity available for strategic learning.

**Fig. 1:**
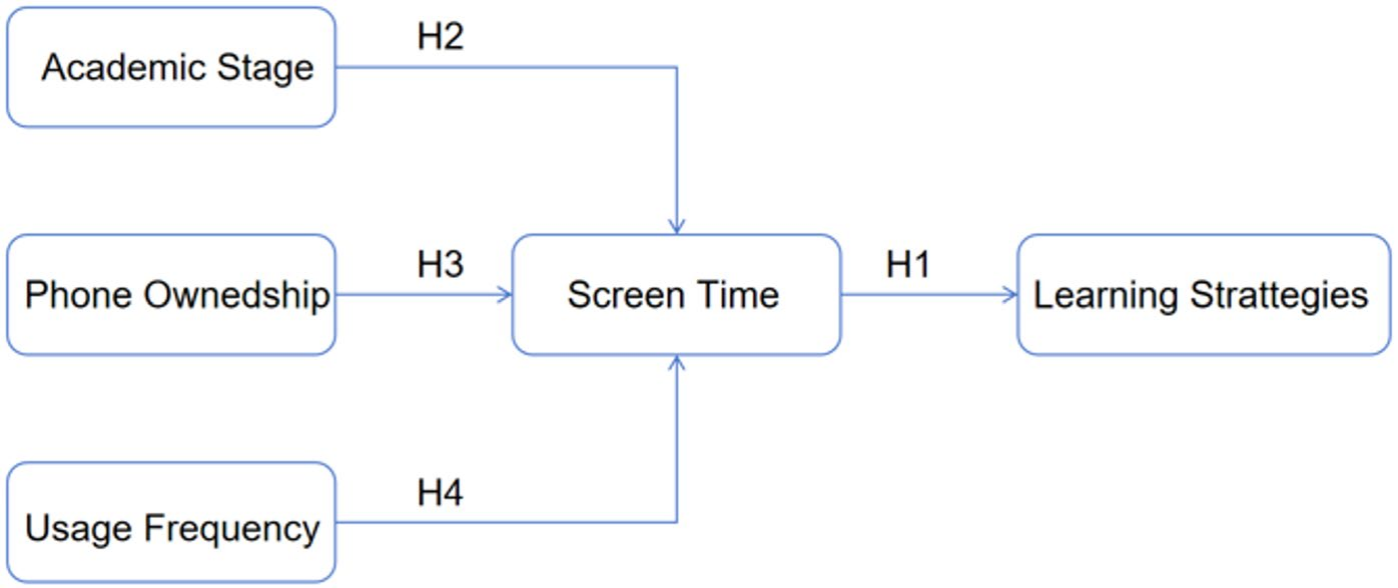
Theoretic Hypotheses Framework.

### Research hypotheses

Based on our analytical framework (Fig. 1), we test:

H₁: Daily short-video screen time will negatively correlate with overall learning strategy scores (β < 0), with strongest effects on metacognitive strategies.

H₂: High school students will exhibit stronger negative associations than middle school students, reflecting developmental sensitivity to digital interference.

H₃: Phone owners will show steeper learning strategy declines per screen-hour than non-owners, indicating access-driven overuse.

H₄: Screen time and learning strategies will demonstrate a nonlinear relationship, with accelerated decline beyond 2 h/day.

## Participants and methods

### Inclusion and exclusion criteria and questionnaire exclusion

1. Inclusion criteria: ① Middle school and high school students; ② Understand the purpose and content of this study, agree to participate in the research and cooperate in answering the questions; ③ Can scan the QR code or click on the questionnaire link on the computer to answer the questions.
2. Exclusion criteria: ① Those who were absent from school due to various reasons during the investigation period; ② students who repeated grade12.
3. Questionnaire exclusion criteria: ① Incompleted questionnaire filling and blank basic information; ② the time used was less than 300 s; ③ consistent answers to 10 or more consecutive questions in the questionnaire.

### Questionnaire

This study was a quantitative validation study; data were collected by questionnaire developed from past theories and research. The general information section collected fundamental demographic data, as well as participants’ attitudes and opinions regarding short-videos. The assessment of learning strategies utilized Zhang Yeheng’s “Learning Strategy Scale“^7^. The scale comprises 45 items, each rated on a 5-point Likert scale (from 1 = strongly disagree to 5 = strongly agree). The higher the score, the greater the perception of the content of the variable. The Cronbach’s alpha reliability coefficient for the developer’s testing scale was 0.869, while the split-half reliability coefficient was 0.8598.

### Data collection

Eighteen schools from six administrative districts around Shijiazhuang in HeBei province were selected through cluster sampling, questionnaires were delivered through QR codes to students from the first year of middle school to the third year of high school in Jan 2025. Following telephone agreement from the guardian, the informed consent form was included in the survey link. Participants who agreed to informed consent completed the questionnaire, while those who disagreed departed the survey link.

All methods in this study were performed in accordance with the relevant guidelines and regulations for research involving human subjects, and the study protocol was approved by the Research Ethics Committee of the Second Hospital of HeBei Medical University (No.2024-R758).

### Statistical analysis

Data from Questionnaire Star, an online survey platform using QR codes, was exported to Excel and analyzed with SPSSAU software. Incomplete questionnaires and entries with consistent responses were excluded from the analysis. No significant outliers were identified in the final dataset that required further treatment. A descriptive statistical analysis was conducted on the general dataset. Initially, the quantitative data, comprising total scores from the learning strategy scale and scores from each dimension, were assessed for normality. Data with normal distribution were represented as means and standard deviation, and those with non-normal distribution as median and interquartile range (IQR). Categorical variables were summarized using frequency counts and percentages. In instances where the variance between groups was not homogeneous, comparisons were made using non-parametric tests. Differences between two groups were analyzed using the *MannWhitney* test and *Kruskal Wallis* test was used when comparing more than two groups. The test level was *α* = 0.05, and *P* < 0.05 indicated statistical significance.

## Results

### Participants

The number of participants included in this study was 4515 and after removing invalid questionnaires, 3028 valid questionnaires remained, with a valid return rate of 67.07%. The sample included 1371 (45.28%) males and 1657 (54.72%) females. The age of the participants rang from 13 to 19 years old (Table 1).

**Table 1:** Demographic characteristics of participants (*N* = 3028).

Items	Levels	Counts	Percentage(%)
Gender	Male	1371	45.28
	Female	1657	54.72
Years-old	13–15	1744	57.6
	16–19	1284	42.4
Academic levels	Middle School	1818	60.04
	High School	1210	39.96
Personality self-evaluation.	Typical Introverted	383	12.65
	Introverted dominate	1503	49.63
	Extroverted dominate	992	32.75
	Typical Extroverted	150	4.97

### Reason and interest in Short-videos

#### Reasons for watching and browsing

The result indicated that the top reasons for watching short-videos were decompression and relaxation (77.79%), interesting content (63.1%), and information acquisition (48.22%).

**Table 2:**
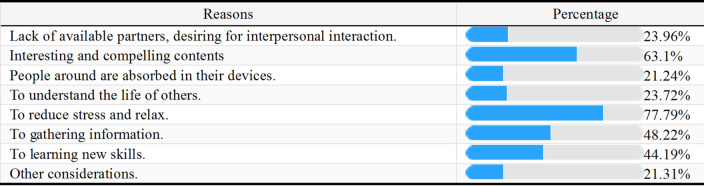
Reasons for Watching Short-videos

#### Participants’ perception of the impact of short-videos

It can be seen that short-videos contribute a lot on participants’ attitude towards life, followed by expression styles and learning methods while relatively small impact on celebrity choices.

**Table 3:**
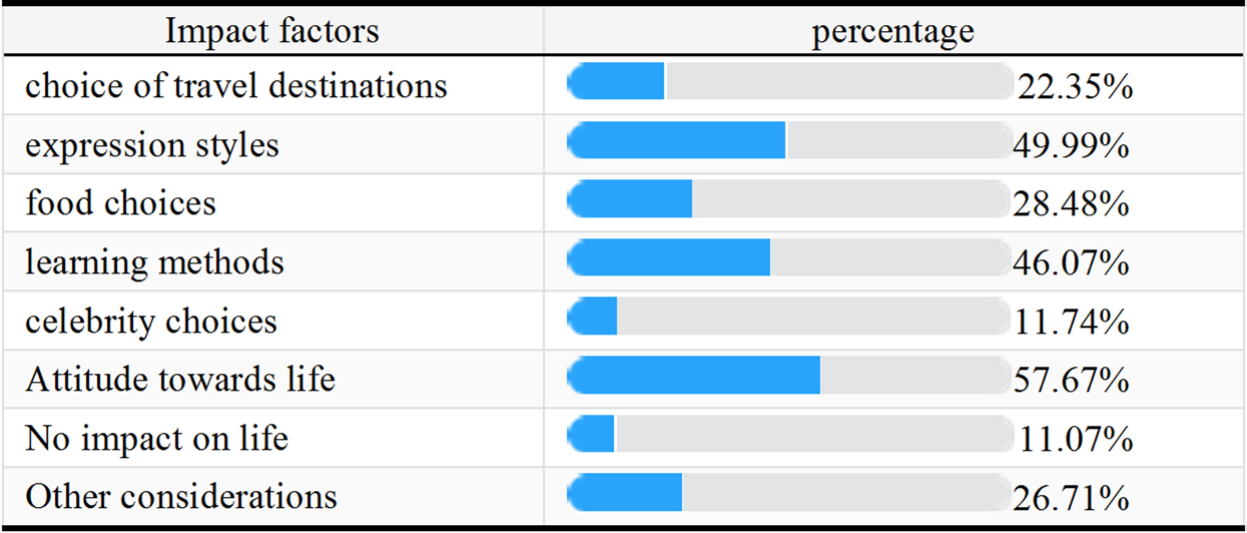
Participants’ Perception of the Impact of Short-videos

#### Ranking the impact of short-videos

The respondents believe that watching short-videos on mobile phones has the greatest impact on vision, followed by its impact on learning, and then self-control. The impact on mood, posture, and personality is relatively small. The specific data is shown in the table below:

**Table 4:** Ranking of the impact of mobile Short-videos from participants’ Perception.

Items	No 1	No2	No 3	No 4	No 5	No 6
vision	1985(47.24%)	412(9.8%)	288(6.85%)	229(5.45%)	250(5.95%)	181(4.31%)
learning	902(22.65%)	936(23.51%)	404(10.15%)	286(7.18%)	219(5.5%)	303(7.61%)
Self control	694(17.78%)	591(15.14%)	521(13.35%)	352(9.02%)	386(9.89%)	841(21.54%)
mood	369(9.79%)	540(14.32%)	906(24.03%)	600(15.91%)	548(14.53%)	467(12.38%)
posture	122(3.24%)	543(14.42%)	528(14.02%)	578(15.35%)	550(14.6%)	619(16.44%)
personality	119(3.23%)	441(11.97%)	578(15.69%)	1018(27.63%)	768(20.85%)	457(12.4%)

### Reliability, Validity, and scores of the learning strategy questionnaire

In the current study, the reliability coefficient of the scale data for this group of subjects is 0.951, and the KMO and Bartlett’s test KMO values are 0.980. The score description of the Learning Strategy Scale is shown in Table 6. The normality test results show that the total score of learning strategies, cognitive strategies, metacognitive strategies, and resource utilization strategies do not have normality traits, so the median description is used.

**Table 5:** Scores of learning strategy Scale.

	25 quanti	median	75 quanti	standard error	95% CI(LL)	95% CI(UL)	IQR	Kurtosis	Skewness	(CV)
Total score	133.000	143.000	159.000	0.452	144.994	146.765	26.000	0.866	0.213	17.037%
cognitive strategy	44.000	47.000	53.000	0.165	48.000	48.649	9.000	0.642	0.224	18.837%
Metacognitive Strategy	41.000	45.000	50.000	0.149	45.416	45.999	9.000	0.745	0.124	17.896%
Resource utilization strategy	47.000	51.000	57.000	0.164	51.527	52.168	10.000	0.978	0.230	17.353%

### Analysis of learning strategy scores by subject characteristics

#### Learning strategies at different learning stages and different ages

**Table 6:** Non parametric testing of learning strategy scores at different academic Stages.

	M(P25, P75)		U value in MannWhitneytest	z value in MannWhitney	*p*
	Middle school (*n* = 1818)	High school (*n* = 1210)			
cognitive strategy	48.000(44.0,54.0)	46.000(43.0,52.0)	961714.000	−5.871	0.000**
Metacognitive Strategy	45.000(41.0,51.0)	44.000(41.0,49.0)	1003568.500	−4.092	0.000**
Resource utilization strategy	52.000(48.0,58.0)	50.000(46.0,55.0)	922539.000	−7.533	0.000**
Total score	145.000(134.0,162.0)	140.000(130.0,154.0)	954815.000	−6.158	0.000**

* *p* < 0.05 ** *p* < 0.01.

As shown in Table 7, middle school students demonstrated significantly higher median scores than high school students in cognitive strategies, metacognitive strategies, and resource utilization strategies (all *p* < 0.01). Consequently, the total learning strategy score was also significantly higher for middle school students.

In addition, Kruskal Wallis test analysis showed that there was a significant statistical difference (*P* = 0.003) in the total score of learning strategies among samples of different ages, which is consistent with the conclusion of differences in learning stages.

#### Learning strategies in different genders

The Mann Whitney test analysis showed that there was no significant difference in the total score of learning strategies, metacognitive strategy dimension, and resource utilization strategy score between male and female samples of different genders (*p* > 0.05), indicating that there was no difference in the scores of learning strategies, metacognitive strategy dimension, and resource utilization strategy between male and female participants. There was a difference in cognitive strategy scores between male and female participants (*p* = 0.016).

**Table 7:** Non parametric test of the learning strategies in different Genders.

	M(P25, P75)		U value MannWhitney	z value MannWhitney	*p*
	Male(*n* = 1371)	Female(*n* = 1657)			
Cognitive strategy	47.000(43.0,53.0)	47.000(44.0,53.0)	1078078.000	−2.416	0.016*
Metacognitive Strategy	44.000(41.0,51.0)	45.000(41.0,50.0)	1097902.000	−1.588	0.112
Resource utilization strategy	51.000(47.0,57.0)	51.000(47.0,56.0)	1133255.500	−0.109	0.913
Total score	142.000(131.0,158.0)	143.000(133.0,159.0)	1099554.000	−1.517	0.129

* *p* < 0.05 ** *p* < 0.01.

#### Owning your own phone and learning strategy score

**Table 8:** Non parametric test of owning mobile phone and learning Strategy.

	M(P25, P75)		U value MannWhitney	z value MannWhitney	*p*
	YES(*n* = 2187)	NO(*n* = 841)			
Cognitive strategy	47.000(43.0,52.0)	49.000(45.0,57.0)	751609.500	−7.807	0.000**
Metacognitive Strategy	44.000(41.0,49.0)	46.000(42.0,53.0)	778097.000	−6.576	0.000**
Resource utilization strategy	50.000(46.0,55.0)	53.000(48.0,60.0)	737471.500	−8.462	0.000**
Total score	141.000(132.0,155.0)	148.000(135.0,170.0)	745092.000	−8.102	0.000**

* *p* < 0.05 ** *p* < 0.01.

From the above table, it can be seen that the Mann Whitney test analysis shows that the total score and scores of each subscale of learning strategies for subjects who have their own mobile phones are significantly higher than those who do not have their own mobile phones, *P* = 0.000.

#### Time selection and learning strategy score for mobile phone browsing

**Table 9:** Non parametric test of time selection and learning strategy Scores.

	M(P25, P75)			H value Kruskal-Wallis	*p*
	Mon to Fri(*n* = 27)	weekend(*n* = 2800)	Everyday(*n* = 201)		
Cognitive strategy	47.000(40.0,54.0)	47.000(44.0,53.0)	45.000(40.0,49.0)	34.930	0.000**
Metacognitive Strategy	45.000(42.0,49.0)	45.000(41.0,51.0)	42.000(38.0,47.5)	35.116	0.000**
Resource utilization strategy	48.000(45.0,53.0)	51.000(47.0,57.0)	48.000(43.0,52.0)	45.799	0.000**
Total score	139.000(128.0,155.0)	143.000(133.0,160.0)	135.000(123.0,146.0)	44.144	0.000**

* *p* < 0.05 ** *p* < 0.01.

From the above table, it can be seen that the distribution of time spent watching mobile videos varies, and the scores of learning strategies also show significant differences with *P* = 0.000. The total scores of learning strategies and resource utilization strategies for weekend mobile phone users are significantly higher than those for daily mobile phone users and those who only use mobile phones from Monday to Friday. And the total scores of learning strategies, cognitive strategies, and metacognitive strategies for daily mobile phone users were significantly lower than those for weekend mobile phone users, *P* = 0.000.

#### Screen time and learning strategy score

**Table 10:** Non parametric test of screen time and learning strategy Score.

	Average screen time everyday in the last week (hr per day)						H value Kruskal-Wallis	*p*
	Around 1 h (*n* = 1500)	Around 2 h (*n* = 679)	Around 3 h (*n* = 462)	Around 4 h (*n* = 194)	Around 5 h (*n* = 72)	Over 5 h (*n* = 121)		
Cognitive strategy	49.000	47.000	45.000	45.000	43.500	42.000	202.627	0.000**
Metacognitive Strategy	46.000	44.000	43.000	43.000	41.000	41.000	160.653	0.000**
Resource utilization strategy	53.000	51.000	49.000	48.000	47.500	46.000	209.078	0.000**
Total score	149.000	142.000	137.000	136.500	133.000	130.000	212.760	0.000**

* *p* < 0.05 ** *p* < 0.01.

From the above table, it can be seen that there are significant differences in the total scores of cognitive strategies, metacognitive strategies, resource utilization strategies, and learning strategies among samples with different durations of mobile phone usage (average daily time in the past week), with *P* = 0.000. The total scores of learning strategies, cognitive strategies, and metacognitive strategies for those who spend more time on mobile phones are significantly lower than those who spend less time on mobile phones.

## Discussion

### Short video usage in adolescent

By August 2024, the number of short video users in China reached 1.1 billion according to the 54th Statistical Report on China’s Internet Development, with 7.42 million new Internet users, mainly among teenagers aged 10–19 and the elderly group. Among them, teenagers accounted for 49.0% of the new Internet users^8^. In our study, 72.22%(2187/3028) participants have their own cellphone, and 6.7% (201/3028) watch cellphone everyday (Tables 9 and 10). Short-videos have become an important application for the new Internet users to “connect to the Internet”. This surge in users reflects a broader trend in the digital landscape, where platforms like TikTok and Kuaishou have become integral to daily life, particularly among younger demographics. Our results provides empirical support for the Resource Model of Self-Control, suggesting that the cognitive resources depleted by prolonged and frequent short-video consumption are the same finite resources necessary for employing effective metacognitive and resource management strategies.

#### Reasons for watching short-videos

As participants in the current investigation reported (Table 2), *relieve stress and relax* are the most frequently used reasons for browsing the short-videos, followed by the *fun and video contents*. This indicates that the popularity of short-videos among teenagers is closely related to the platforms’ ability to provide instant gratification and stress relief. Short video platforms can quickly capture user interests and provide personalized content through algorithmic recommendation systems, thereby attracting and retaining special users. For instance, a study on digital advertisements found that tailoring content to specific demographics, such as age and gender, resulted in a notable increase in click-through rates, indicating that users are more likely to engage with content that resonates with their personal characteristics^9^. Moreover, the integration of machine learning techniques in video summarization and recommendation systems has been shown to improve user interactions by curating content that aligns with individual interests. This approach not only addresses the challenges of managing vast amounts of multimedia data but also ensures that users receive relevant content tailored to their viewing habits^10^. Therefore, the more viewers seek entertainment and stress-relieving content, the more they are exposed to such video content. With the popularity of short video content, viewers’ engagement with visually appealing content also demonstrates an impact on their self-image and emotions, which may lead to more frequent consumption of such content^11^. As a result, content marketing is driven not only by the audience’s viewing history, but also by their psychological and emotional state, thus the reasons for watching short-videos may also influence the content to which they are typically exposed.

#### Effects of short-videos on participants’ perceptions

In the current study, the impact of short-videos was presented as a question allowing for multiple answers. According to the result showed in Table 4, the top three options with the highest click-through rates are life attitude, expression style, and learning style. In the ranking question (Table 5), the participants considered that the most significant effect of watching short-videos on smartphones is on eyesight, with learning and self-discipline being the next most affected areas. The influence on mood, posture, and personality is comparatively minor. Prolonged exposure to screens can lead to various visual problems, including digital eye strain, which is characterized by symptoms such as dryness, irritation, and difficulty focusing^12^. This is particularly concerning for children and adolescents, who may be more susceptible to these effects due to their developing eyes^13^.

Additionally, research on Intelligent English Education based on short video recommendation algorithms demonstrated that such systems can enhance the efficiency of English learning by tailoring content to user preferences. This aligns with findings that suggest short-videos can cater to different learning styles, as seen in the varied responses to video versus live lecture formats, where students expressed preferences based on their learning needs. Moreover, the role of short-videos in shaping life attitudes and expressions among learners has been examined. Research indicates that various media formats, including educational videos, can significantly influence learners’ perceptions and emotional responses. For instance, the impact of presenter gender and video format on public comment sentiment has been explored, revealing that videos featuring female presenters often elicit stronger emotional reactions compared to those with male presenters^14^. This suggests that the characteristics of video content can shape not only viewer engagement but also their attitudes towards the subject matter.

Moreover, the nature of content consumed through short-videos can influence learning outcomes. For instance, educational video games have been shown to enhance motivation and learning in children, suggesting that the format of short-videos can be leveraged for educational purposes^15^. The integration of interactive elements in video content can further enhance engagement and retention of information, promoting self-discipline as learners navigate through educational materials^16^. The use of mobile technologies, including smartphones, has transformed traditional learning environments, allowing for more flexible and personalized learning experiences. However, this shift also necessitates the development of self-regulated learning strategies among students to manage their screen time effectively and mitigate potential negative impacts on their eyesight and overall well-being^17^. These findings substantiate the Resource Model of Self-Control, as the fragmented nature of short-video consumption continuously depletes adolescents’ finite cognitive resources, thereby compromising their capacity to implement and sustain effective learning strategies as our analysis showed.

## Participants’ learning strategies

### Learning strategy score

The academic performance of adolescents is often assessed through various measures, including grades, test scores, and overall engagement in school activities. Zhang Yeheng’s Learning Strategy Scale^7^, specifically designed for middle school students within the Chinese educational system, encompasses three distinct dimensions: the cognitive strategy dimension, the metacognitive strategy dimension, and the resource management and utilization strategy dimension, which integrates all the aforementioned factors. The cognitive strategy dimension encompasses paraphrasing, elaboration, and organizational strategies; the metacognitive strategy dimension includes planning, goal-setting, and monitoring strategies; and the resource management and utilization strategy dimension involves time management, learning environment utilization, effort regulation, and help-seeking strategies. The scale also demonstrated excellent reliability and validity in this study (Table 6).

Studies have shown mixed results regarding the correlation between screen time and academic achievement^18^. Some research suggests that excessive screen time, particularly from recreational activities, can detract from time spent on educational tasks, thereby negatively impacting academic performance. Conversely, screen time utilized for educational purposes may enhance learning opportunities and engagement, thereby supporting academic success.

In the current investigation, our results showed that middle school students generally score higher than high school students in cognitive strategies, metacognitive strategies, and resource utilization strategies. This may be related to the greater academic pressure and time management challenges faced by high school students. The effective application of learning strategies is crucial for improving learning efficiency and academic performance^19^. By understanding the strategies students use, educators can identify specific learning needs and tailor interventions accordingly. Insights from the scale can help in developing programs that enhance effective learning strategies, ultimately improving academic performance.

### Academic stage and learning strategies

The result in Table 7 showed that middle school participants generally score higher than high school participants in various dimensions of learning strategies. This may be related to the fact that middle school students have more time and energy invested in exploring and applying learning strategies compared to high school students. This study excluded vocational high school students, who can choose to enter the workforce instead of taking college entrance exams. Consequently, the only path for regular high school students in China is to pass the exams, gain university admission. Thus, the academic pressure of high school students may result in their less proactive application of learning strategies compared to middle school students^19^.

### Gender and learning strategies

The survey result from Table 8 showed that gender has no significant impact on learning strategies, but boys score higher than girls in cognitive strategies. This may be related to gender differences in cognitive processing and information processing. Research has shown that men tend to perform better in spatial cognition and analysis tasks, while women perform better in language and memory tasks^20^. However, these gender differences do not necessarily mean that one gender has inherent advantages in learning, but are influenced by socio-cultural factors and educational environments.

### Mobile phone ownership in the investigated participants

Data from Table 9 showed that participants who do not own their own mobile phones have significantly higher scores compared to those who have their own mobile phonesin learning strategies. This phenomenon can be attributed to several factors, including the accessibility of educational resources, the ability to engage in mobile learning, and the integration of technology into daily study habits. For instance, smartphone applications designed for educational purposes can enhance learning by providing interactive content and facilitating communication with peers and educators^21^. Furthermore, the implementation of adaptive e-learning platforms has been shown to boost student engagement and perceived knowledge, particularly in fields requiring complex understanding^22^. These findings underscore the potential of technology, when used thoughtfully, to enhance academic performance and encourage effective learning strategies. Furthermore, the convenience of mobile technology allows for flexible learning environments, enabling students to study anytime and anywhere, thus fostering a more personalized approach to education^23,24^. In conclusion, the ownership of mobile phones appears to be a significant factor influencing learning strategies among students. The integration of mobile technology, ipad for example, into educational practices not only supports academic achievement but also enhances social interactions and collaborative learning experiences^24^.

### Time selection for flashing mobile phones

The result in Table 11 showed that the longer the participants use their mobile phones, the lower their learning strategy scores. This provide further evidence for the hypothesis that excessive use of mobile phones may have a negative impact on the application of learning strategies. Long term use of mobile phones may lead to distraction, decreased time management ability, and weakened learning motivation. A study highlighted that chronic sensory stimulation from screens can alter brain structures, particularly gray and white matter volumes, which are crucial for cognitive functions such as memory and learning^25,26^. This alteration may result in short-term impairments in attention, memory acquisition, and recall, which share some similarities with symptoms observed in mild cognitive impairment (MCI)^25^. Research has shown that excessive screen time is associated with attention problems in children, highlighting the importance of monitoring screen exposure, especially in the context of increased online learning due to the pandemic^27^. Moreover, attention plays a vital role in visual short-term memory (VSTM), where selective attention can restore forgotten items to memory. This suggests that when attention is compromised, as may occur with excessive screen time, the ability to encode and recall information can also be negatively impacted.

Furthermore, from Table 10 we could see that those participants who used their phones on weekends scored significantly higher in learning strategies than those who used their phones every day. This aligns with research indicating that excessive smartphone use can negatively impact cognitive functions and learning outcomes. For instance, a study found that multitasking with smartphones during academic activities was negatively correlated with students’ grades, indicating that frequent switching between tasks can reduce efficiency and learning outcomes^28^. This aligns with findings that excessive smartphone use can lead to increased levels of perceived stress, which further exacerbates academic challenges and impacts overall mental health^29^.

Moreover, the context in which mobile phones are used plays a crucial role in their impact on learning. A study on parental phone use during mealtimes revealed that distractions from mobile devices could lead to less effective communication and engagement, which are essential for fostering a conducive learning environment for children^30^. This suggests that the timing and manner of phone usage—such as using phones primarily on weekends—may allow for more focused and intentional learning strategies compared to daily usage, which could be more fragmented and distracting. Thus, our results provide robust validation for the primary four research hypotheses.

## Conclusion

Short-videos, as an emerging medium, exert a complex impact on the learning strategies of teenagers.In terms of participants’ perceptions, visual problems were identified as the most significant physical impact. There is a negative correlation between daily short-video screen time and overall learning strategy scores, with the most pronounced effects observed on metacognitive strategies. Participants who engaged with short-videos primarily on weekends demonstrated significantly higher learning strategy scores compared to those who used their phones daily. High school participants experiencing greater academic pressure exhibited less proactive application of learning strategies than middle school participants. Furthermore, phone owners experienced a more pronounced decline in learning strategy scores per screen-hour compared to non-owners, suggesting overuse driven by accessibility. The learning strategy scores of participants who spent more than two hours per day on short-video screen time declined at an accelerated rate. These findings suggest that educators and parents should encourage balanced short-video usage and promote structured screen-time schedules, particularly on weekdays, to better support the development of adolescents’ learning strategies.Therefore, it is necessary for families, schools, and society to work together to guide young people to use short-videos healthily and play a positive role in learning.

The above analysis is based on survey data and combined with relevant research literature to explore in depth the impact of short video usage on adolescent learning strategies. However, the present study is subject to several limitations. Firstly, the use of cluster sampling may have constrained the geographical scope, thereby limiting the generalizability of the findings. The sample did not include students from rural areas or adolescents outside the traditional middle/high school setting, such as vocational high school students, which further restricts the representativeness of the sample in relation to the broader adolescent population. Additionally, the cross-sectional quantitative design precludes the establishment of causal relationships, making it unclear whether excessive short video usage leads to lower learning strategy scores or if students with inherently lower learning strategy scores are more inclined to engage in such video consumption. Furthermore, all data were collected through self-reported questionnaires, which may result in participants either overestimating or underestimating their screen time. Lastly, the study tool, Zhang Yeheng’s “Learning Strategy Scale,” developed in 2007, may not adequately capture contemporary learning strategy dimensions, potentially leading to incomplete measurement.

## Data Availability

The raw anonymized dataset generated and analyzed during the current study is available in the WenJuanXing repository, https://www.wix.cn/report/267016779.aspx.
